# Biomarkers of Inflammation in Left Ventricular Diastolic Dysfunction

**DOI:** 10.1155/2019/7583690

**Published:** 2019-06-02

**Authors:** Mihaela Mocan, Larisa Diana Mocan Hognogi, Florin Petru Anton, Roxana Mihaela Chiorescu, Cerasela Mihaela Goidescu, Mirela Anca Stoia, Anca Daniela Farcas

**Affiliations:** ^1^“Iuliu Hatieganu” University of Medicine and Pharmacy, Department of Internal Medicine, Cluj-Napoca, Romania; ^2^Emergency Clinical County Hospital, Department of Internal Medicine, Cluj-Napoca, Romania; ^3^Emergency Clinical County Hospital, Department of Cardiology, Cluj-Napoca, Romania

## Abstract

Left ventricular diastolic dysfunction (LVDD) is an important precursor to many different cardiovascular diseases. Diastolic abnormalities have been studied extensively in the past decade, and it has been confirmed that one of the mechanisms leading to heart failure is a chronic, low-grade inflammatory reaction. The triggers are classical cardiovascular risk factors, grouped under the name of metabolic syndrome (MetS), or other systemic diseases that have an inflammatory substrate such as chronic obstructive pulmonary disease. The triggers could induce myocardial apoptosis and reduce ventricular wall compliance through the release of cytokines by multiple pathways such as (1) immune reaction, (2) prolonged cell hypoxemia, or (3) excessive activation of neuroendocrine and autonomic nerve function disorder. The systemic proinflammatory state causes coronary microvascular endothelial inflammation which reduces nitric oxide bioavailability, cyclic guanosine monophosphate content, and protein kinase G (PKG) activity in adjacent cardiomyocytes favoring hypertrophy development and increases resting tension. So far, it has been found that inflammatory cytokines associated with the heart failure mechanism include TNF-*α*, IL-6, IL-8, IL-10, IL-1*α*, IL-1*β*, IL-2, TGF-*β*, and IFN-*γ*. Some of them could be used as diagnosis biomarkers. The present review aims at discussing the inflammatory mechanisms behind diastolic dysfunction and their triggering conditions, cytokines, and possible future inflammatory biomarkers useful for diagnosis.

## 1. Introduction

Left ventricular diastolic dysfunction (LVDD) is a preclinical condition defined as the inability of LV to fill an adequate end-diastolic volume (preload volume) at an acceptable pressure [1]. LVDD is an important precursor to many different cardiovascular diseases. It represents the dominant mechanism (2/3 of patients) in the development of heart failure (HF) with preserved ejection fraction (HFpEF), which shows a rising prevalence in older population (by 2020, more than 8% of people over 65 are estimated to have HFpEF) and is associated with a poor prognosis [2]. Diastolic abnormalities have been studied extensively in the past decade, and it has been confirmed that chronic low-grade inflammatory reaction is the key mechanism leading to HF [3].

A new paradigm of LVDD development was recently proposed. Classical cardiovascular risk factors, grouped under the name of metabolic syndrome (MetS), or other systemic diseases that have an inflammatory substrate such as chronic obstructive pulmonary disease (COPD), atrial fibrillation (AF), anemia, or chronic kidney disease (CKD) induce myocardial structural and functional abnormalities through low-grade systemic and endothelial inflammation (IF). IF triggers oxidative stress (OS) cascade in the coronary microvascular endothelial cells and reduces nitric oxide (NO) bioavailability in the myocardial cells. Following NO decreased availability, myocardial cyclic guanosine monophosphate- (cGMP-) protein kinase G (PKG) signaling is reduced, causing maladaptive hypertrophy and increased cardiomyocyte stiffness [4].

The newly published joined European and American guidelines underline the diagnosis difficulties of LVDD [5], as echocardiographic measurements are considered partly nonsensitive or inconclusive [6]. Therefore, it is of utmost importance to find biomarkers and risk scores that enable us to have an early diagnosis and enhance the prognosis of HF patients.

Baring these in mind, the present review aims at discussing the inflammatory mechanisms behind LVDD and their triggering conditions, cytokines, and possible future inflammatory biomarkers useful for diagnosis.

## 2. Pathological Mechanisms of Left Diastolic Dysfunction

The diastole is the part of the cardiac cycle that includes the isovolumetric relaxation phase and the filling phases and has passive and active components. The filling of the LV is divided into rapid filling during early diastole, diastasis, and rapid contraction phase during the late contraction phase. LVDD can be the consequence of abnormalities during any phase of the diastole. Thus, impaired relaxation, high filling pressure, increased LV operating stiffness, mechanical asynchronism, increased peripheral artery stiffness, and the loss of atrial contraction at higher heart rates are just some of the underlying mechanisms in LVDD [7].

Patients with LVDD are generally older, more often female, and have a high prevalence of CVD and other morbid conditions, such as obesity, metabolic syndrome, diabetes mellitus type 2, salt-sensitive hypertension, atrial fibrillation, COPD, anemia, and/or renal dysfunction. Each one of these pathologies were proved to be linked to LVDD and could lead to LVDD through different pathways.

The incidence of LVDD associated to HFpEF is increasing with *global aging*. LVDD, left atrial remodeling, and cardiac fibrosis along with vascular changes such as endothelial dysfunction, arterial stiffening, and vascular IF are all the attributes of the advanced age [8]. The effect of aging on ECM was nicely synthesised by Meschiari et al. [9]. In brief, senescence modifications of the cardiovascular system increase afterload and impair vasodilation, which increases LV's wall stress leading to cardiomyocyte hypertrophy. Hypertrophic cardiomyocytes have increased oxygen needs, and the imbalance between supply and demand of oxygen favors reactive oxygen species (ROS) production with toxic effect on cardiomyocytes. In response to hypoxemia, cardiomyocytes release proinflammatory cytokines and chemokines promoting IF and recruiting macrophage in the LV [10]. Macrophages are a rich source of matrix metalloproteinases (MMP) which are linked to myocardial aging status and LVDD. Moreover, aging favors amyloid deposit in LV, which increases myocardial thickening, described as senile amyloidosis. The possible mechanism is still under debate but may be linked to posttranscriptional biochemical alterations of transthyretin or its chaperones [11].

*Metabolic syndrome* (*MetS*) has been associated with LVDD with preserved systolic function. With cardiovascular risk factors clustered in the MetS, as triggers, IF favors pathological changes in the myocardium leading to relaxation abnormalities [12].

The key mechanism responsible for LVDD in MetS patients is not entirely understood. In animal models with diet-provoked MetS, the hypertrophy and fibrosis of the myocardial cells were caused by accelerated OS. In mouse models of dyslipidemia, high blood pressure, or insulin resistance IF, along with endothelial dysfunction played an important role in the development of cardiac fibrosis and increased myocardial stiffness [13].

In previous studies, our group demonstrated that IF biomarkers have a good predictive potential for LVDD [14, 15] showing a strong association between LVDD and IL-6 levels, independent of MetS components and NT-proBNP. Thus, IL-6 could be useful in identifying asymptomatic patients with MetS and LVDD and applying lifestyle measures to prevent overt heart failure development. Others have reported an association between IF biomarkers and LVDD in patients with symptomatic heart failure [16], and studies on animal models showed that pathological elevations of IL-6 [5] result in extensive cardiac fibrosis, by regulating cell function through a cell surface receptor. Our results come to add knowledge to this two-step model of LVDD in MetS patients by pointing out IL-6 as the IF biomarker with the best predictive capacity for LVDD.

The systemic proinflammatory state present in *chronic obstructive pulmonary disease* (*COPD*) patients might contribute to vascular and myocardial abnormalities leading to an increased risk of cardiovascular morbidity, especially during acute exacerbations. López-Sánchez et al. demonstrated that a systemic inflammatory pattern characterized by increasing IL-6 and CRP was associated with LVDD in a homogeneous population of severe stable COPD patients [6]. Development of LV alterations manifested through LVDD is found in more than 90% of the subgroup of severe COPD patients, independently of age and the presence of systemic hypertension [7]. The IF was present, mostly in sedentary and obese patients, and could be more closely related to obesity or lower physical activity than to the degree of airway obstruction. On the other hand, extracellular matrix (ECM) proteins such as MMP can act as IF stimuli by modulating the proinflammatory response of the heart, synthetizing cytokines and growth factors. In patients with myocardial injuries such as ischemia, myocarditis, and advanced heart failure, tenascin-C (Tn-C), an ECM glycoprotein, was transiently expressed in myocardial tissue, in association with immediate tissue repair response and the final deposit of collagen in the damaged tissue [17].

The role of *chronic kidney disease* (CKD) in the development of LVDD was elegantly summarized recently by ter Maaten et al. [18]. In brief, CKD causes metabolic and systemic abnormalities in circulating factors, inducing an activated systemic IF (CRP, TNF-*α*, IL-6, sST2, and pentraxin-3) and microvascular dysfunction (favored by chemokines, adhesion molecules, and cytokines), which may lead to cardiomyocyte stiffening, hypertrophy, and interstitial fibrosis via cross-linking between the microvascular and cardiomyocyte compartments [19]. As for common biomarkers, galectin-3 has proved its utility in identifying both early CKD [20] and incident cardiac fibrosis [21].

A high prevalence of *atrial fibrillation* (AF) in association with LVDD and HFpEF (up to 60%) is reported by numerous studies (CHARM programme, ADHERE Core, and SwedeHF) [22, 23]. This could potentially be explained by shared pathological conditions (MetS, obesity, hypertension, coronary artery diseases, and atrial myocardial injury) promoting low-grade systemic IF and leading to simultaneous development of AF and LVDD [24]. The same mediator molecules are found in both AF and LVDD: CRP, TNF-*α*, IL-6, IL-8, IL-10, IL-1*α*, IL-1*β*, IL-2, TGF-*β*, and IFN-*γ*, along with MMP and ROS [19].

Several neurohormonal and mechanistic hypotheses have been proposed for the IF-LVDD continuum: (1) the activation of the renin-angiotensin-aldosterone system (RAAS) stimulating the production of proinflammatory cytokines (such as IL-6, IL-8, and TNF-*α*), directly activating immune cells and increasing the expression of adhesion molecules such as vascular cell adhesion protein 1, intercellular adhesion molecule 1, selectins, or MCP-1 and (2) elevated LV diastolic pressure might induce cardiac apoptosis, and OS, which can subsequently induce regional IF thereby increasing production of IL-1, IL-6, and TNF-*α* [19].

*The neurohormonal hypothesis of RAAS* activating OS was verified by Negi et al. in a well-performed clinical study [25], trying to explain the negative results from RAAS inhibitor therapy in HFpEF patients. The authors found that HFpEF was not associated with RAAS activation or systemic OS [25]. On the other hand, preclinical studies showed that angiotensin-II induces mitochondrial dysfunction, OS, reducing eNOS bioavailability and impairing myocardial relaxation [26]. Some possible explanations are available so far. First of all, OS may take place only in the affected myocardium (OS “signaling is compartmentalized”) explaining the absence of systemic OS markers in patients with HFpEF [27]. Secondly, OS in the myocardium may appear earlier than systemic OS. At last, other mechanism may be responsible of LVDD progression, given the polymorphism of etiological and trigger factors.

The *activation of mineralocorticoid receptors* through aldosterone may be an important factor in the pathogenesis of HFpEF through multiple mechanisms such as cardiac fibrosis or endothelial dysfunction [1, 28]. In this respect, mineralocorticoid receptor agonists (MRA) have been studied in patients with HFpEF or ischemic HFpEF (after myocardial infarction). Although in some of the studies MRA failed to improve mortality in HFpEF (such as the TOPCAT trial), others showed that MRA could improve LVDD and reduce cardiac remodeling having positive impact on the quality of life. These studies were analyzed by Chen et al. [29] in an extensive meta-analysis which concluded that “MRA treatment may exert beneficial effects, including reduced hospitalizations due to HFpEF, improved life quality and diastolic function, and cardiac remodeling reversal, without an effect on all-cause mortality.” These are indirect evidence that RAAS is implicated in pathogenesis of LVDD and HFpEF.

Another mechanism proposed in LVDD was myocardial *microvascular dysfunction* [30]. Mohammed et al. performed in 124 myocardial autopsy specimens of patients with HFpEF. The authors found out that microvascular density and myocardial fibrosis are more frequent in patients with HFpEF and are not related to the severity of epicardial coronary stenosis, supporting the hypothesis of microvascular endothelium IF in LVDD pathogenesis. Moreover, there was an inverse relation between fibrosis and microvessel density [31]. In this respect, Kato et al. conducted an imagistic study (cardiac magnetic resonance (CMR)) and calculated the coronary flow reserve (CFR) in hypertensive patients with LVDD. They proved that CFR was decreased in these patients and correlated significantly with NT-proBNP values. Both pathological and imagistic data indicate that myocardial microvascular impairment might contribute to the development and progression of LVDD [32]. Despite the evidence of microvascular dysfunction, the therapy aiming vasodilation (*angiotensin-converting enzyme inhibitors*, *angiotensin-II receptor blockers*, and *phosphodiesterase-5 inhibitors*) that had had promising results in experimental studies yielded negative or neutral results in large clinical trials.

Thus, a meta-analysis of the clinical trials of *angiotensin-converting enzyme inhibitors and angiotensin-II receptor blockers* (CHARM-Preserved, I-Preserve, and PEP-CHF) showed no effect of these drugs on mortality or hospitalization rate in patients with HFpEF. The beta-blocker and spironolactone trials arrived at neutral conclusions [33]. The potential effects of *phosphodiesterase-5 inhibitors* were assessed in a randomized, double-blind, placebo-controlled clinical trial of 216 patients with stable HFpEF who showed no improvement in exercise capacity or clinical status, after 8 months [34].

With regard to *molecular basis* of LVDD, the data about IF are scarce. Westermann et al. investigated LVDD mechanisms by performing endomyocardial biopsy samples and analyzing the inflammatory cells and their inflammatory products, in vitro. The authors elegantly showed that CD3-, CD11a-, and CD45-marked inflammatory cells had higher concentrations in LVDD myocardial tissue as compared with controls. Moreover, the VCAM-1 adhesion molecule and TGF-*β*, along with oxygen radical production, were found to be increased in LVDD patients but with no significant change in serum concentration of CRP [3].

Any mechanism that interferes with actin-myosin crossbridge detachment, intracellular changes in titin or microtubules, extracellular changes in collagen, and infiltration was proved to be responsible for LVDD [35].

Recent studies on both animal and human models showed that titin isoform shift, ROS, nitric oxide synthetase (NOS) dysfunction that results in decreased nitric oxide (NO), and myosin-binding protein C (MyBP-C) are implicated in LVDD [35]. Increased titin N2B isoform expression and the reduced phosphorylation of titin were linked to elevated cardiomyocyte stiffness in endomyocardial biopsy samples of patients with LVDD [2]. ROS resulted from OS, and *advance glycation end products* cause LVDD in diabetic patients [36]. Jeong et al. showed in an experimental mouse model that high-fat diet leads to mitochondrial ROS production and LVDD through insulin resistance and glucose intolerance. The mitochondria-targeted antioxidant administration to the high-fat diet mouse model prevented LVDD development and progression [37]. This study proved that mitochondrial OS actively participates to development and progression of LVDD, and its inhibition represents a potential therapy target. In this same study, low-carb diet or glycemic control was unable to reverse LVDD [38].

In clinical settings, a meta-analysis showed an obvious trend of reduction in mortality rates in *HMGCoA reductase inhibitor* users from 2005 to 2013, as a consequence of their pleiotropic and antioxidant effects [39], supporting the hypothesis that HMGCoA reductase inhibitors may improve survival in HFpEF [40].

Advanced glycation end products (AGEs) result from glucose interactions with proteins via nonenzymatic ways and accumulate in a variety of pathological conditions such as hypertension or diabetes mellitus [41]. AGE accumulation in the myocardium was found in patients with diabetes mellitus [42]. Serum concentrations of some AGE might be predictive for mortality and hospitalization rates in HFpEF patients [41]. Thus, AGE became a potential therapeutic target. Alagebrium is a cross-link breaker that showed promising results in small studies but discouraging conclusions in larger ones [43].

NOS is an important modulator of cardiac nitroso-redox balance and function. Uncoupled NOS in hypertensive mouse models results in decrease in NO that are consistent with increased cytosolic calcium and LVDD [44]. In human studies, G894T polymorphism of the eNOS gene and MetS was related to arterial stiffness and can be a connection pathway between MetS and the increased cardiovascular risk [45]. Finally, *MyBP-C* is a thick protein localized in the striated muscle sarcomeres, and it plays an important role in cardiac contraction and relaxation. Experimental studies showed that phosphorylation of MyBP-C leads to impaired cardiac muscle contraction and subsequent LVDD [36]. Further, cMyBP-C decrease LV remodeling in response to pressure overload [46]. Thoonen et al. identified MyBP-C as a cGMP-dependent protein kinase I leucine zipper (PKGI*α* LZ) binding partner and kinase substrate, with great importance for the possible therapeutic targets in HFpEF [47]. The experimental study of Jeong et al. showed that preventing glutathionylation of MyBP-C using cofactor tetrahydrobiopterin ameliorates diastolic dysfunction through reversing changes of myofilaments [48]. These data “provide evidence that cardiac relaxation could be modified by posttranslational changes of myofilament proteins.” In clinical studies, MyBP-C had both diagnosis and prognosis properties in patients with HFpEF. Tong et al. observed that cMyBP-C is a potential screening biomarker for the existence of severe cardiovascular diseases [49]. Jeong et al. considered it as a novel biomarker in HF patients, with the capacity to discriminate between HFpEF, having higher values than in HFrEF (4.02 ± 1.4 vs. 2.01 ± 0.61) [50].

**Figure 1:**
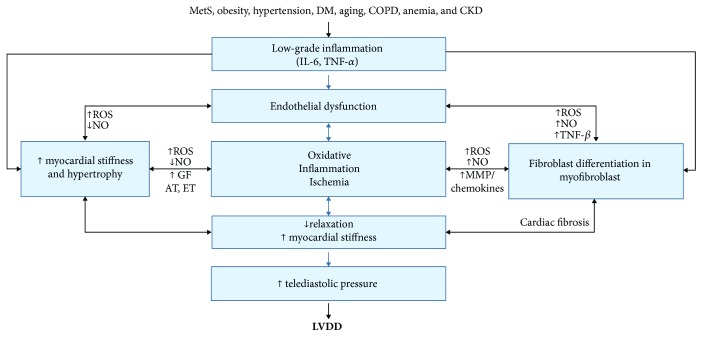
Scheme showing the interrelation between trigger conditions and LVDD via systemic IF (adapted after von Bibra et al. [52]). IL-6: interleukin-6; hsCRP: high-sensitivity C reactive protein; ROS: reactive oxygen species; NO: nitric oxide; MMP: matrix metalloproteinases; GF: growth factors; AT: angiotensin; ET: endothelin; TNF-*β*: tumor necrosis factor beta.

At last, fibroblasts differentiate into myofibroblasts and secrete collagen into ECM. Shifts in the collagen type (from type III to type I) could impair the cardiac biomechanism by contributing to increased LV stiffness [9]. These mechanisms are synthetized in Figure 1.

Given these data, we can state that IF is an important link in the pathogenesis of LVDD, and thus, it is conceivable that treatments targeting IF will require the development of new treatment modalities in patients with LVDD. Studies using targeted immunomodulating therapy in HF were elegantly reviewed by [51].

## 3. Inflammatory Biomarkers for Diastolic Dysfunction

In this pathological chain, activation of persistent immune response is currently considered to stay at the origin of inflammatory cytokine secretion. In LVDD with or without HFpEF, the current hypothesis is that the associated conditions (described above) are the triggers to immune reaction with the production of a vast amount of proinflammatory cytokines. These cytokines could be a measurement of the risk of LVDD development rather than quantification of severity [53]. In HF patients, on the other hand, IF biomarkers are closely associated with pathogenesis, poor functional state, and adverse prognosis.

Natriuretic peptides, especially N-terminal pro-BNP (NT-proBNP), have been extensively studied as a diagnosis biomarker of HFpEF, showing lower cut-off values than those in HFrEF [54]. In the ESC guideline (2016) for the diagnosis of HFpEF, along with echocardiographic criteria, the elevations in BNP or NT-proBNP are recommended for the identification of elevated LV filling pressures. Moreover, the guideline stipulates that “the negative predictive values are very similar and high (94-98%) in both the nonacute and acute settings, but the positive predictive values are lower both in the nonacute setting (44-57%) and in the acute setting (66-67%).” To this point, the ESC's guidelines recommend that the diagnosis of HFpEF should be based on structural and Doppler findings of LVDD, and elevated NT-proBNP should be used to rule out HF [55]. Even though, at the moment, NT-proBNP represents a standard biomarker for HFpEF, one can only wonder whether it is trustful enough for the positive diagnosis in HFpEF. The initial results from the large registries such as DIAST-CHF (Diastolic Congestive Heart Failure) which showed a sensitivity of 65% for the diagnosis of HFpEF only increased mistrust and stimulate the search for other biomarkers to increase diagnostic accuracy [56]. In contrast to brain natriuretic peptides, inflammatory biomarkers used independently or associated with multimarker scores raise high expectations both for positive diagnosis and prognosis of HFpEF [4, 57].

Proinflammatory cytokines involved in LVDD (both with and without HFpEF) are interleukins (IL-6, IL-8, IL-10, IL-11, IL-1*α*, IL-1*β*, and IL-2), tumoral necrosis factors (TNF-*α*, TGF-*β*), and interferon (IFN-*γ*). Other biomarkers quantifying IF in LVDD are MCP-1, galectin-3, sST2, and GDF-15.

### 3.1. CRP, IL-6, IL-8, IL-11, and TNF-*α*

CRP, TNF-*α*, and IL-6 were among the first to be described as having multiple sites of action both on the vascular endothelium and at the myocyte level, where they enhance apoptosis, inducing hypertrophy or dilation [53]. Additionally, cytokine levels in LVDD are the result of a complex dysregulation of the cytokine. This could include activation of mediators involved in both IF and myocardial fibrosis such as IL-6, as well as a lack of overall regulation of the immune response by impaired function of regulatory T cells [51].

*CRP* is considered a biomarker of diagnosis and severity rather than a key player in LVDD. Michowitz et al. showed that hsCRP was higher in patients with LVDD and HFpEF, as compared with healthy patients. Moreover, in these patients, levels of hsCRP correlated with NYHA class (and therefore the severity of HFpEF), and the main predictors of hsCRP levels are NYHA class and diabetes mellitus [58]. In the study performed by our group, hsCRP proved to be a predictive marker for LVDD in MetS patients [14].

*IL-6* is playing a central role in IF initiation and progression in cardiovascular diseases [59]. IL-6 infusion in rats results in LV hypertrophy, increased collagen volume fraction, and increased myocardial stiffness. Studies have shown that IL-6 could be linked to the increased number of major cardiac events and cardiomyocyte hypoxic stress [60]. In our study, IL-6 proved to be an independent predictive biomarker for LVDD in MetS patients [14]. IL-6 and hsCRP proved to be biomarkers of prognosis in MetS associated with LVDD [61]. Moreover, increased levels of IL-6 correlate with the severity of HF and are strongly prognostic of 1-year mortality [62].

*IL-8* has been demonstrated to increase the expression and production of osteopontin, which stimulates interstitial fibrosis, and TGF-*β*, which stimulates collagen synthesis, and inhibit matrix degradation by reducing MMP. Collier et al. have shown that IL-8 and MCP-1 [57] also play a role in the development and worsening of LVDD as it has been shown in different studies [63].

*IL-11* has pleomorphic actions and is capable of upregulating or downregulating inflammatory processes according to different states of the microenvironment [64]. One of the mechanisms through which IF induces LVDD is fibrosis. This is a common process in the pathology of cardiovascular disease, and it seems that IL-11 targets cardiac myocytes thorough pathways that could either protect or be deleterious for them. Also, research has shown that fibroblasts express IL-11 required for the synthesis of fibrogenic proteins. Research has shown that fibroblast expressing IL-11 was responsible for fibrosis, but deletion of IL-11RA_1_ provided protection against this condition [65]. Aside from the effect on myocardial fibrosis, the other pathways through which it acts are still unknown and under research.

A study which observed patients with CAD showed that IL-11 was mainly secreted by macrophages and may be related to cardiac atherosclerotic disease initiation and progress, being found in high concentration in plasma and aorta of patients with aortic dissection [66]. If we focus on the effects of IL-11 on patients with HF, studies have shown that its plasma concentrations are significantly increased and related to the severity of HF and to the number of cardiovascular events. Furthermore, bearing in mind its protective effects, IL-11 might become a new target for the therapy and prevention in HF patients [64].

*TNF-α* induces myocardial apoptosis and myocardial stiffness, playing a major role in the progression of LVDD. The myocardial apoptosis is a consequence of activating p38 mitogen-activated protein kinase, stimulating iNOS to transform NO to ONOO-, and of increased ROS synthesis. Myocardial stiffness is aggravated by the imbalance of MMP activity, with an increased ratio of MMP/TIMP and changes in collagen fibers, favored by TNF-*α* secretion [67]. The increased production and reduced degradation of collagen and increased activation of lysyl oxidase-1, resulting in a cross-linked and insoluble collagen network, may in turn result in LVDD. In another study performed by our group, LVDD in coronary disease patients did not show a good correlation with TNF-*α* levels but with leptin levels [68]. TNF-*α* was reported to have both an involvement in cardiac dysfunction and a protective effect on ischemic myocardium. The expression of the two TNF-*α* receptors might be responsible for TNF-*α* conflicting actions, and ischemic myocardium remodeling is a consequence of the balance between TNF-*α* actions [68]. Dunlay et al. in the Olmsted County study found that mortality in HF patients is directly correlated with TNF-*α* and not influenced by EF value [69]. Thus, TNF-*α* could be useful for the prognosis of LVDD. TNF-*α* receptors (sTNFR1 and sTNFR2) were found to be higher in HFpEF patients [51].

Furthermore, assessing these cytokines in large populations of well-characterized patients may provide insight information regarding the pathophysiology of LVDD. Unfortunately, cytokines circulate at low levels, thus requiring high-sensitivity assays and large population studies, which represents the main disadvantage of using them as biomarkers for LVDD. More reliable biomarkers could be the corresponding soluble receptors of soluble ligands which are frequently detected in high concentrations in serum and plasma [51].

### 3.2. Pentraxin-3

Pentraxin-3 belongs to a superfamily of proteins together with CRP and serum amyloid-associated protein, but it differs from the latter through the monomer constitute. Pentraxin-3 has five long monomers, and their role is primarily at the interface of the immune system IF and ECM [70]. There are several types of cells that produce pentraxin-3: immune system cells such as mononuclear cells and neutrophils and also adipocytes, fibroblasts, and smooth muscle cells. In one study they conducted, Matsubara et al. proved that pentraxin-3 is produced in the coronary circulation in patients with LVDD. When they compared patients with HFpEF to healthy individuals, they found a direct and positive correlation between pentraxin-3 and LVDD. Pentraxin-3 was produced in the coronary circulation in patients with LVDD. Furthermore, pentraxin-3 levels were higher than those of hsCRP, IL-6, or TNF-*α* levels in patients with LVDD [71]. Even though pentraxin-3 proved to be a good diagnosis biomarker, assessing pentraxin-3 in relation to LVDD prognosis was not established until recently. The same group showed that high plasma pentraxin-3 levels, but not other inflammatory markers, are correlated with future cardiovascular events in patients with HFpEF. The authors concluded that pentraxin-3 may be a useful biomarker for assessment of risk stratification in HFpEF [72]. The impossibility of this biomarker to distinguish HFpEF of HFrEF is an important pitfall.

### 3.3. MCP-1

Monocyte chemoattractant protein 1 (MCP-1) has been shown in animal studies to be required for macrophage infiltration, the induction of TGF-*β* and the development of reactive fibrosis, and LVDD progression [73]. Cardiomyocyte-targeted expression of MCP-1 in mice caused death by heart failure at 6 months of age. MCP-induced protein expression increased in parallel with the development of ventricular dysfunction. In situ hybridization showed that the presence of MCP-induced protein transcripts in the cardiomyocytes was associated with apoptosis [74]. MCP-1 could be a potential therapeutic target as gene therapy with an MCP-1 antagonist was recently found to attenuate the development of ventricular remodeling in a mouse model for ischemic HF [51].

In human studies, MCP-1 was increased, along with other IF biomarkers (IL-6, IL-8) in hypertensive patients with LVDD, without proving to be an independent diagnosis marker or prognosis factor [57]. Additional research in ischemic HF patients showed that both lower and higher MCP-1 levels are associated with an increased risk of all-cause and cardiovascular mortality [75], but further research is need to confirm these findings.

### 3.4. Galectin-3

Galectin-3 is a beta-galactosidase binding lectin, with a wide variety of biological functions in IF, immunity, and cancer. It has recently been proposed to be a novel biomarker of LVDD. It was found to be involved in cell adhesion, growth, and differentiation, but also, it is involved in the process of fibroblast activation with known chemoattractant and proapoptotic roles [51].

The axis galectin-3/cardiotrophin-1 (Gal-3/CT-1) was found to be one of the mechanisms through which these properties are manifested. Martínez-Martínez et al. found in a study completed on Wistar rats that once treated with CT-1, they presented a higher cardiac Gal-3 level and a higher degree of myocardial fibrosis and also perivascular fibrosis. They concluded that an elevation of both molecules in HF patients could mean higher cardiovascular mortality and that the axes CT-1/Gal-3 might become a therapeutic target and also a HF biomarker [76]. Other data suggests that Gal-3 could also enhance a pathway through myocardial fibrosis, by activating RAAS. This might have therapeutic aim in the near future [77].

In HF patients, Gal-3 may be a biomarker of poor prognosis related to excessive and potentially irreversible myocardial fibrosis, which again may be related to enhanced IF. In this respect, a comprehensive review about the predictive value of Gal-3 was written by Coburn et al., in 2014 [77]. In brief, Gal-3 was repeatedly shown to be elevated in the setting of IF processes underlying HF and proved to be a better prognosis biomarker in HF than other conventional IF markers currently in use, such as natriuretic peptides or hsCRP. Besides that, it is worth mentioning that De Boer et al. showed that predictive value of Gal-3 appeared to be stronger in patients with HFpEF and correlated with echocardiographic measurements of LVDD [78]. Recently, van Vark et al. in the TRIUMPH (Translational Initiative on Unique and Novel Strategies for Management of Patients with Heart Failure) clinical cohort study, composed of 496 acute HF patients, evaluated the levels of circulating Gal-3. Elevated circulating Gal-3 appeared to be a strong predictor of outcome in acute HF patients, independent of N-terminal probrain natriuretic peptide. Hence, galectin-3 may be helpful in the clinical practice for prognostication and treatment monitoring [79].

### 3.5. Soluble ST2

ST2 is a part of the IL-1 receptor family with an important role in regulating IF and immunity. This protein has two isoforms—ST2L which is a receptor and sST2 which responds to myocardial stretching in relation to elevation of filling pressure. When IL-33 binds to sST2-L, it produces a cascade of events that prevent the progression of myocardial hypertrophy and fibrosis. But in contrast, circulating plasma sST2 limits this binding, therefore promoting alterations in the myocardial structure [80]. Given the apparent contribution of static and pulsatile hemodynamic overload to the pathophysiology of LVDD, sST2 may be a particularly relevant marker of diagnosis, disease progression, and prognosis [54].

Our group showed, recently, a positive and strong correlation between the LV mass and severity of LVDD and the plasma level of sST2, in hypertensive patients with LV hypertrophy. The pathogenic hypothesis in this case is that sST2 might be also produced by the vascular endothelial cells as a consequence to the diastolic load. Another interesting observation is that the increased plasma level of ST2 performed better in predicting LV hypertrophy in hypertensive patients, than NT-proBNP [81]. Moreover, ST2 levels were correlated with the risk of adverse cardiovascular outcome in hypertensive patients with LVDD and increased filling pressure and may represent a useful prognostic marker in these patients [82]. The studies regarding sST2 predictive capacity in HF have been elegantly synthetized by Dattagupta and Immaneni [83], by Bayés-Genís et al. [84], and very recently by Dieplinger and Mueller [85]. In summary, there are large studies such as the Pro-BNP Investigation of Dyspnea in the Emergency Department (PRIDE) study, PROTECT study, SHOP study, and Val-HEFT study showing that ST2 values predict prognosis in chronic HF patients and over time were significantly and independently associated with mortality [83]. ST2 has been recently added in the American College of Cardiology/American Heart Association guidelines with a class II indication for the prognosis in HF [86].

As LVDD and HFpEF are a complex syndrome, only one biomarker is not enough for the diagnosis and prognosis. Thus, a HF risk calculator has been recently developed, the Barcelona bioHF, which comprises sST2 along with brain natriuretic peptide and troponin, to stratify the mortality risk and hospitalization within 5 years. Another score focusing on remodeling and sudden death in HF patients is the ST2-R2 score, which includes sST2 along with several other clinical parameters and which has a high accuracy in predicting reverse remodeling of LVDD [84]. Unfortunately, the aspect of how determining sST2 correlates with LVDD and HFpEF in order to become a diagnosis or prognostic biomarker is still not entirely known. However, ST2 levels appeared to be lower in decompensated HFrEF than in HFpEF, even though not related to 1-year mortality [87].

### 3.6. GDF-15

Growth differentiation factor 15 (GDF-15) was first named macrophage inhibitory cytokine-1 and is a member of the TGF-*β* cytokine superfamily which links it to IF, increased filling pressures, and tissue injury [88]. Under normal, physiological conditions, this hormone is underexpressed, but ischemia-reperfusion injury, oxygen reactive species, and pressure overload upregulate its production. Apparently, GDF-15 plays a protective role in the above conditions by inhibiting apoptosis, hypertrophy, and adverse remodeling via PI3K-Akt, ERK1/2, and SMAD 2/3, thus having a positive impact on the fractional shortening [89]. HF is a condition that was found to be in association with plasma levels of this biomarker, especially in patients with ischemic heart disease [90].

Besides diagnostic capacity, GDF-15 might have screening capacity for unmasking the risk of developing LVDD in a healthy elderly and increasing diagnosis accuracy of asymptomatic LVDD [91]. Thus, Stahrenberg et al. demonstrated that GDF-15 has similar concentrations in both HFpEF and HFrEF. It is independently associated with exercise capacity impairment and quality of life in HFpEF. Diagnostic precision of GDF-15 is at least as good as natriuretic peptide, and the combining signification of NT-proBNP and GDF-15 could increase HFpEF diagnostic accuracy [92]. Moreover, Santhanakrishnan et al. revealed similar results in an Asian population, concluding that GDF-15 distinguished HFpEF patients at least as well as NT-proBNP and the combination of both the biomarkers, providing a useful screening and diagnosis tool for LVDD [93]. Later on, Chan et al. performed a similar study on a large Asian population—Singapore Heart Failure Outcomes and Phenotypes (SHOP) study—and proved that GDF-15, unlike NT-proBNP, was similarly elevated in both types of HF. Thus, GDF-15 has additional prognostic utility over NT-proBNP and hsTnT in both HFpEF and HFrEF. Moreover, serial measurements of GDF-15 provided additional predictive information for outcomes, making GDF-15 a reliable prognosis and risk stratification biomarker [94].

The information regarding novel IF biomarkers in LVDD or HFpEF are synthetized in Table 1. Unfortunately, most studies have sought for prognosis biomarkers in HF rather than diagnosis biomarkers for LVDD; thus, the information regarding specificity and sensibility for the diagnosis of LVDD or HFpEF is not available in all the cited studies. Some authors focused on the correlation between IF biomarker concentrations and echocardiographic criteria for LVDD, while others sought the differences between HFrEF and HFpEF. Moreover, some of the cited studies have small sample size and lack full adjustment. Furthermore, some of the studied biomarkers are at low levels, thus increasing analytical variation and requiring expensive high-sensitivity assays that should be tested on large sample population. Larger trials are clearly needed to obtain pathophysiological information. A future meta-analysis of previous data regarding the diagnosis role of IF biomarkers in HFpEF could be of help to deconvolute markers of HF in general from markers of isolated LVDD.

## 4. Conclusions and Future Trends

LVDD or impaired ventricular relaxation is one of the multiple mechanisms underlying the complex syndrome of HFpEF. Multiple comorbidities are the triggers of LVDD progression to HFpEF. LVDD diagnosis is nowadays based solely on echocardiography, even though it is characterized by multiple pathogenic factors and is associated with a plethora of biomarkers. In the future, the association of these three diagnosis tools (clinical identification of comorbidities, echocardiography, and IF biomarkers) in risk scores that could allow patients' risk stratification and detection of LVDD in early asymptomatic phases would reduce significantly the burden of HFpEF.

Many of the IF biomarkers are currently under investigation. Until now, they did not enter the clinical practice and had similar or lower diagnosis and prognosis capacity as compared to natriuretic peptides. Further research is needed to identify the most reliable biomarker for the early diagnosis, progression monitoring, and prognosis in patients with LVDD.

The development of molecular target immunotherapy that enhances ventricular-vascular coupling, cardiomyocyte stiffness at the level of the myofilaments, or other inflammatory and immunopathogenic pathways could have a benefit in preventing LVDD progression to HFpEF.

## Figures and Tables

**Table 1:** Novel inflammatory biomarkers for diagnosis and/or prognosis in LVDD and HFpEF.

Biomarker	Authors	Clinical study	Population (*n*)	Diagnosis biomarker	Prognosis biomarker
Single marker					
*CRP*	Sciarretta et al. [95]		128	Correlated with LVMI and *E*/*E*′	
	Koller et al. [96]	LURIC study	459		HR: 1.32 (95% CI 1.08–1.62), CV mortality at 5 years
	Sinning et al. [97]	GHS study	5000	AUC 0.66 (95% CI: 0.61–0.71)	HR: 1.5 (95% CI: 1.3–1.7)
	DuBrock et al. [98]	RELAX study	214	Higher levels in LVDD	
*IL-6*	Haugen et al. [62]		72	Higher levels in LVDD	Cut − off value > 10 ng/L, 1-year mortality
	Mocan et al. [14]		72	AUC 0.73 (95% CI: 0.61–0.83)	
	Kloch et al. [99]	EPOGH study	303	Correlated with *E*′ (*r* = 0.039)	
*IL-8*	Collier et al. [57]		275	Higher values in HFpEF hypertensive patients	
	Phelan et al. [100]		41	Higher levels with greater LVMI and LAVI	
*TNF-α*	Sciarretta et al. [95]		128	Correlated with LVMI and *E*/*E*′	
	Dunlay et al. [69]	Olmsted County study	486		HR: 2.10 (95% CI: 1.30–3.38)
*Pentraxin-3*	Matsubara et al. [71]		82	OR: 1.49 (95% CI: 1.11-1.98)	
*MCP-1*	Ding et al. [75]	Guangdong Coronary Artery Disease Cohort	1411		HR: 1.5-2.11 C-index +12,6%
*Galectin-3*	Shah et al. [101]	PRIDE study	115	Correlated with *E*/*E*′ (*r* = 0.035)	
	De Boer et al. [78]	COACH study	592		HR: 1.97 (1.62–2.42), better for HFpEF than for HFrEF
	Edelmann et al. [102]	Aldo-DHF trial	422		HR: 3.319 (95% CI: 1.214-9.07), all-cause death or hospitalization at 6 or 12 months
*Soluble ST2*	Bartunek et al. [103]		163	ST2 mARN higher in LVDD, correlated with LVEDP	
	Shah et al. [104]		134	Correlated with *E* amplitude	
	Manzano-Fernández et al. [105]		447		Cut-off 0.35 ng/mL HR: 3.26 (95% CI: 1.50–7.05), prediction of 1-year mortality
	Shah et al. [106]		387		HR: 2.85 (95% CI: 2.04–3.99), prediction of 1-year mortality
	Santhanakrishnan et al. [93]	SHOP study	151	Cut-off 26.47 ng/mL, AUC 0.662 (95% CI: 0.554–0.770) Se 70%, Sp 48% for HFpEF	
	Wang et al. [107]			Cut-off 13.5 ng/mL OR: 11.7 (95% CI: 2.9-47.4) for HFpEF	
	Anand et al. [108]	VAL-HEFT study	1650		Cut − off sST2 ≤ 33.2 ng/mL Cox logHR: 0.048 (0.031-0.065), 1-year mortality
	Sinning et al. [97]	GHS study	5000	AUC 0.62 (95% CI: 0.56–0.67)	HR: 1.4 (95% CI: 1.2–1.6)
	Farcas et al. [82]		76		OR: 2.43 (95% CI: 1.32-7.24) at baseline predicts the CV events for 1 year
	Farcas et al. [81]		88	Cut-off 28.14 ng/mL (Se 94.4%, Sp 69.1%) for LVDD Cut-off 14 04 ng/mL (Se 82.1%, Sp 53.8%) for LVH	AUC: 0.732 (95% CI: 0.613–0.850)
	Najjar et al. [109]		193		HR: 6.62 (95% CI: 1.04–42.28) for mortality or rehospitalization
*GDF-15*	Stahrenberg et al. [92]		1935	Cut-off 1.16 ng/mL, AUC 0.891 (95% CI: 0.850-0.932)	
	Santhanakrishnan et al. [93]	SHOP study	151	Cut-off 879 pg/mL (Se 92%, Sp 84%) Cut-off 1120 pg/mL (Sp 92%, Se 82%)	
	Sinning et al. [97]	GHS study	5000	AUC 0.79 (95% CI: 0.75–0.83)	HR: 1.7 (95% CI: 1.6–1.9)
	Chan et al. [94]	SHOP study	488		HR: 1.68 (95% CI: 1.15–2.45) CV events at 6 months
*MyBP-C*	Jeong et al. [50]			Higher values in HFpEF than in HFrEF (4.02 ± 1.4 vs. 2.01 ± 0.61)	
	Tong et al. [49]		158		Prestress cut-off 127 ng/mL, HR: 8.1 (95% CI: 1.09-60.09) Poststress cut-off 214 ng/mL, HR: 4.77 (95% CI: 1.75-12.98)

Multimarker score					
*CRP+GDF-15+sST2/NT-proBNP and GDF-15/NT-proBNP*	Sinning et al. [97]	GHS study	5000	Discrimination between HFpEF and HFrEF	
*NT-proBNP+GDF-15*	Stahrenberg et al. [92]		1935	AUC 0.942 (0.912-0.972) GDF − 15 ≥ 1.16 ng/mL + NT − proBNP ≥ 200.7 ng/L (Se 56.6%, Sp 98.9%)	
	Chan et al. [94]		488	AUC: 0.891 (95% CI: 0.850-0.932) for GDF-15	HR: 1.68 (95% CI: 1.15–2.45), risk for composite outcome (mortality and rehospitalization)

AUC: area under the curve; CI: confidence interval; CRP: C reactive protein; CV: cardiovascular; EPOGH: European Project on Genes in Hypertension; GDF-15: growth differentiation factor 15; GHS: Gothenburg Heart Study; IL: interleukin; HFrEF: heart failure with reduced ejection fraction; HFpEF: heart failure with preserved ejection fraction; HR: hazard ratio; LAVI: left atrial volume index; LVDD: left ventricular diastolic dysfunction; LVED: left ventricular end-diastolic pressure; LVMI: left ventricular mass index; MCP-1: monocyte chemoattractant protein 1; MyBP-C: myosin-binding protein C; NT-proBNP: N-terminal probrain natriuretic peptide; OR: odds ratio; PRIDE: Pro-BNP Investigation of Dyspnea in the Emergency Department; RELAX: Phosphodiesterase-5 Inhibition to Improve Clinical Status and Exercise Capacity in Diastolic Heart Failure; SHOP: Singapore Heart Failure Outcomes and Phenotypes; TNF-*α*: tumor necrosis factor alpha; sST2: soluble ST2; VAL-HEFT: Valsartan Heart Failure Trial.
